# Vogesella oryzagri sp. nov., isolated from the rhizosphere of rice and in silico genome mining for the prediction of biosynthetic gene clusters

**DOI:** 10.1099/ijsem.0.006687

**Published:** 2025-02-26

**Authors:** Md. Amdadul Huq, Yeon-Ju Kim, M. Mizanur Rahman, Md. Morshedul Alam, Sathiyaraj Srinivasan, Kwon-Kyoo Kang, Shahina Akter

**Affiliations:** 1 Department of Food and Nutrition, College of Biotechnology and Natural Resource, Chung-Ang University, Anseong-si, Gyeonggi-do, 17546, Republic of Korea; 2 Graduate School of Biotechnology and College of Life Science, Kyung Hee University, Yongin-si, Gyeonggi-do 17104, Republic of Korea; 3 Department of Biotechnology and Genetic Engineering, Faculty of Biological Science, Islamic University, Kushtia 7003, Bangladesh; 4 Department of Biochemistry and Microbiology, School of Health and Life Sciences, North South University, Dhaka 1229, Bangladesh; 5 Department of Bio & Environmental Technology, College of Natural Science, Seoul Women’s University, Seoul, 01797, Republic of Korea; 6 Department of Horticultural Life Science, Hankyong National University, Anseong-si, Gyeonggi-do, 17579, Republic of Korea; 7 Department of Food Science and Biotechnology, Gachon University, Seongnam, 461-701, Republic of Korea

**Keywords:** biosynthetic gene clusters, digital DNA–DNA hybridization, genome sequence, *in silico* genome mining, *Vogesella oryzagri*

## Abstract

A Gram-stain-negative, aerobic, rod-shaped, motile and flagellated novel bacterial strain, designated MAHUQ-64^T^, was isolated from the rhizosphere of rice. The colonies were observed to be creamy white-coloured, smooth, spherical and 0.5–1.1 mm in diameter when grown on Reasoner’s 2A agar medium for 2 days. Strain MAHUQ-64^T^ was able to grow at 10–40 °C, at pH 5.0–9.5 and in the presence of 0–2.0% NaCl (w/v). The strain was positive for both catalase and oxidase tests. The strain was positive for hydrolysis of l-tyrosine. According to the 16S rRNA gene sequence comparisons, the isolate was identified as a member of the genus *Vogesella* and is closely related to *Vogesella oryzae* L3B39^T^ (98.6% sequence similarity) and *Vogesella facilis* TTM-24^T^ (98.2%). The novel strain MAHUQ-64^T^ has a draft genome size of 3 827 146 bp (22 contigs), annotated with 3612 protein-coding genes, 74 tRNA and 4 rRNA genes. The average nucleotide identity (ANI) and digital DNA–DNA hybridization (dDDH) values between strain MAHUQ-64^T^ and its closest member *V. oryzae* L3B39^T^ were 86.5 and 33.4%, respectively. *In silico* genome mining revealed several biosynthetic gene clusters in the genome of the novel strain MAHUQ-64^T^. The genomic DNA G+C content was determined to be 63.4 mol%. The predominant isoprenoid quinone was ubiquinone-8. The major fatty acids were identified as summed feature 3 (comprising C_16  :  1_*ω*7*c* and/or C_16  :  1_*ω*6*c*) and C_16  :  0_. Based on dDDH, ANI value, genotypic analysis and chemotaxonomic and physiological data, strain MAHUQ-64^T^ represents a novel species within the genus *Vogesella*, for which the name *Vogesella oryzagri* sp. nov. is proposed, with MAHUQ-64^T^ (=KACC 22245^T^=CGMCC 1.19000^T^) as the type strain.

## Introduction

The genus *Vogesella* (type species, *Vogesella indigofera*), first established by Grimes *et al*. [1] and emended by Subhash *et al*. [2] and Sheu *et al*. [3], belongs to the family *Neisseriaceae*, order *Neisseriales* and class *Betaproteobacteria* [4]. At the time of writing, the genus *Vogesella* comprises 12 species with validly published names (https://lpsn.dsmz.de/genus/vogesella). Members of the genus *Vogesella* were isolated from various habitats, including rhizosphere, soil, oxidation pond sediment, spring water, pond water, lake water and freshwater river [5,6]. Members of this genus are Gram-stain-negative, rod-shaped and chemoheterotrophic and possess ubiquinone-8 (Q-8) as the major respiratory quinone, summed feature 3 (comprising C_16 : 1_* ω*7*c* and/or C_16 : 1_* ω*6*c*) and C_16 : 0_ as the predominant fatty acids and DNA G+C contents between 61.2 and 68.8 mol% [1,6]. The association of soil microbes with rice plants has been investigated for decades, and therefore, many novel species in different genera have been isolated from paddy soils, such as ‘*Fluviicola chungangensis*’ [7], *Zoogloea oryzae* [8], *Mucilaginibacter oryzae* [9], ‘*Arthrobacter bangladeshi*’ [10] and *Lysobacter humi* [11]. In the present study, we report a Gram-stain-negative bacterium MAHUQ-64^T^, which was isolated during the characterization of the bacterial diversity in the rhizosphere of rice. The soil attaching to the plant roots, termed as rhizosphere, is a living place for diverse soil micro-organisms, which in turn play a major role in determining plant health. The purpose of this study is to clarify the taxonomic position of strain MAHUQ-64^T^ in detail based on phenotypic characteristics and chemotaxonomic and genotypic analysis. Phylogenetic analyses based on 16S rRNA gene and genome sequences and polyphasic characterization revealed that this isolate belongs to the genus *Vogesella* and represents a novel species. Moreover, the genome sequence of novel strain MAHUQ-64^T^ was analysed for the presence of putative natural product BGCs (biosynthetic gene clusters). The availability of whole genome sequences and synthetic biology-inspired tools/approaches makes it possible to utilize these BGCs to develop new chemicals with new structures, new activity and new targets [12]. Our data revealed that the novel strain MAHUQ-64^T^ contains several BGCs, indicating their potential capability to produce new chemicals with biological activity.

## Isolation and cultivation

During the investigations of bacterial biodiversity, a novel bacterium, designated MAHUQ-64^T^, was isolated from the rhizosphere of a rice field located in Anseong, South Korea. One gram of soil sample was suspended in 9 ml of sterile 0.85% (w/v) NaCl solution. The suspension was serially diluted up to a 10^−6^ dilution, and 200 µl suspension was spread onto Reasoner’s 2A (R2A) agar plates (MB Cell). The plates were incubated at 30 °C for 3 days. Based on colony morphology, five strains (MAHUQ-64^T^, M.A.Huq-107, M.A.Huq-108, M.A.Huq-109 and M.A.Huq-110) were recognized. Single colonies were purified by repeated streaking on fresh R2A agar plates and preserved as a suspension in R2A broth containing glycerol (25%, v/v) at −80 °C. Then, all isolated strains were sent for 16S rRNA gene sequencing. The 16S rRNA gene sequences of all isolated strains have been submitted to the NCBI GenBank database (Table S1, available in the online Supplementary Material). Based on 16S rRNA gene sequence analysis, strain MAHUQ-64^T^ was found as a novel bacterium and selected for detailed taxonomic studies. Strain MAHUQ-64^T^ has been deposited to the Korean Agricultural Culture Collection (KACC) and China General Microbiological Culture Collection Center (CGMCC).

## 16S rRNA gene, genome and phylogenetic analysis

Extraction of the genomic DNA was achieved using a commercial genomic DNA extraction kit (Solgent, Republic of Korea). The 16S rRNA gene was amplified from the chromosomal DNA with the universal bacterial primer pair 27F (5′-AGAGTTTGATCCTGGCTCAG-3′) and 1492R (5′-GGTTACCTTGTTACGACTT-3′) [13], and the purified PCR products were sequenced by Solgent Co., Ltd. (Daejeon, Republic of Korea). The 16S rRNA gene sequences of related taxa were obtained from the GenBank database (http://blast.ncbi.nlm.nih.gov/Blast.cgi) and EzBioCloud server (https://www.ezbiocloud.net) [14]. The multiple sequence alignments were performed by using the clustal_x program [15]. Gaps were edited in the BioEdit program [16]. The evolutionary distances were calculated using the Kimura two-parameter model [17]. The phylogenetic trees were constructed based on 16S rRNA gene sequences using the neighbour-joining (NJ) [18], and the maximum-likelihood (ML) algorithms in the mega 7.0 program [19], with bootstrap values based on 1000 replications. The phylogenetic trees were also constructed using whole-genome sequences based on MLSA (https://automlst.ziemertlab.com/analyze) [20]. In addition, OrthoFinder v2.5.4 (https://github.com/davidemms/OrthoFinder) was used to identify orthologous gene clusters and infer the phylogenetic relationships of the strain with related taxa [21]. The analysis was performed using predicted protein sequences obtained from genome annotation. OrthoFinder implements a graph-based clustering approach to assign genes into orthogroups and reconstruct species and gene trees, providing insights into the evolutionary relationships of the strain [21].

The draft genome sequence of strain MAHUQ-64^T^ was determined using an Illumina HiSeq X Ten and was assembled by SOAPdenovo assembler [22]. The genome annotation was performed by the NCBI prokaryotic genome annotation pipeline [23]. To estimate the degree of pairwise relatedness between MAHUQ-64^T^ and the closest reference strain, blast-based average nucleotide identity (ANI) was calculated as described previously [24]. While the digital DNA–DNA hybridization (dDDH) value was determined using the Genome-to-Genome Distance Calculator (http://ggdc.dsmz.de/ggdc.php) according to Meier-Kolthoff *et al.* [25].

**Fig. 1.**
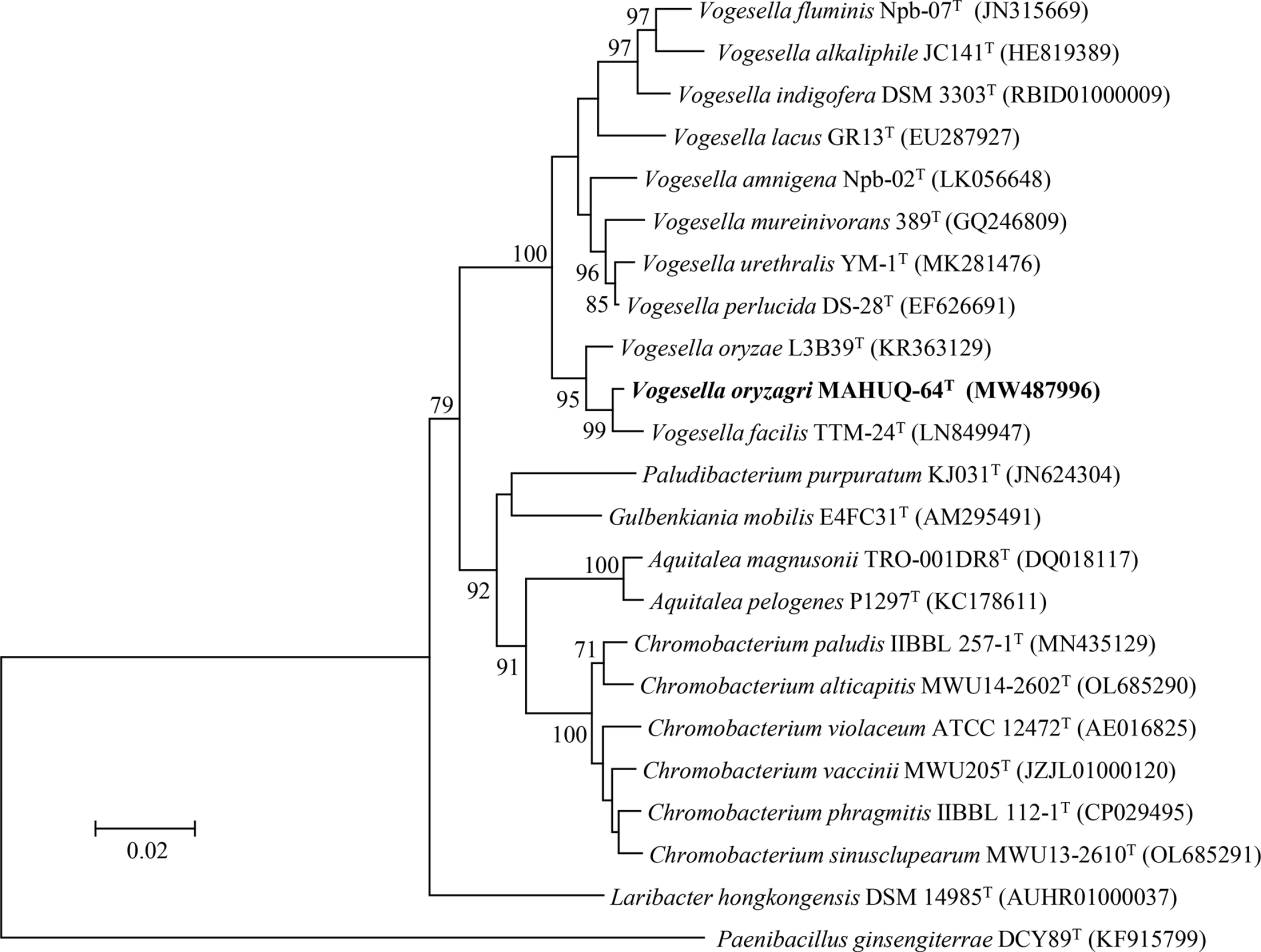
NJ phylogenetic tree based on 16S rRNA gene sequences showing the position of *Vogesella oryzagri* MAHUQ-64^T^ and other related species. Bootstrap values more than 70% based on 1000 replications are shown at branching points. *Paenibacillus ginsengiterrae* DCY89^T^ was used as an outgroup. Scale bar, 0.02 substitutions per nucleotide position.

**Fig. 2.**
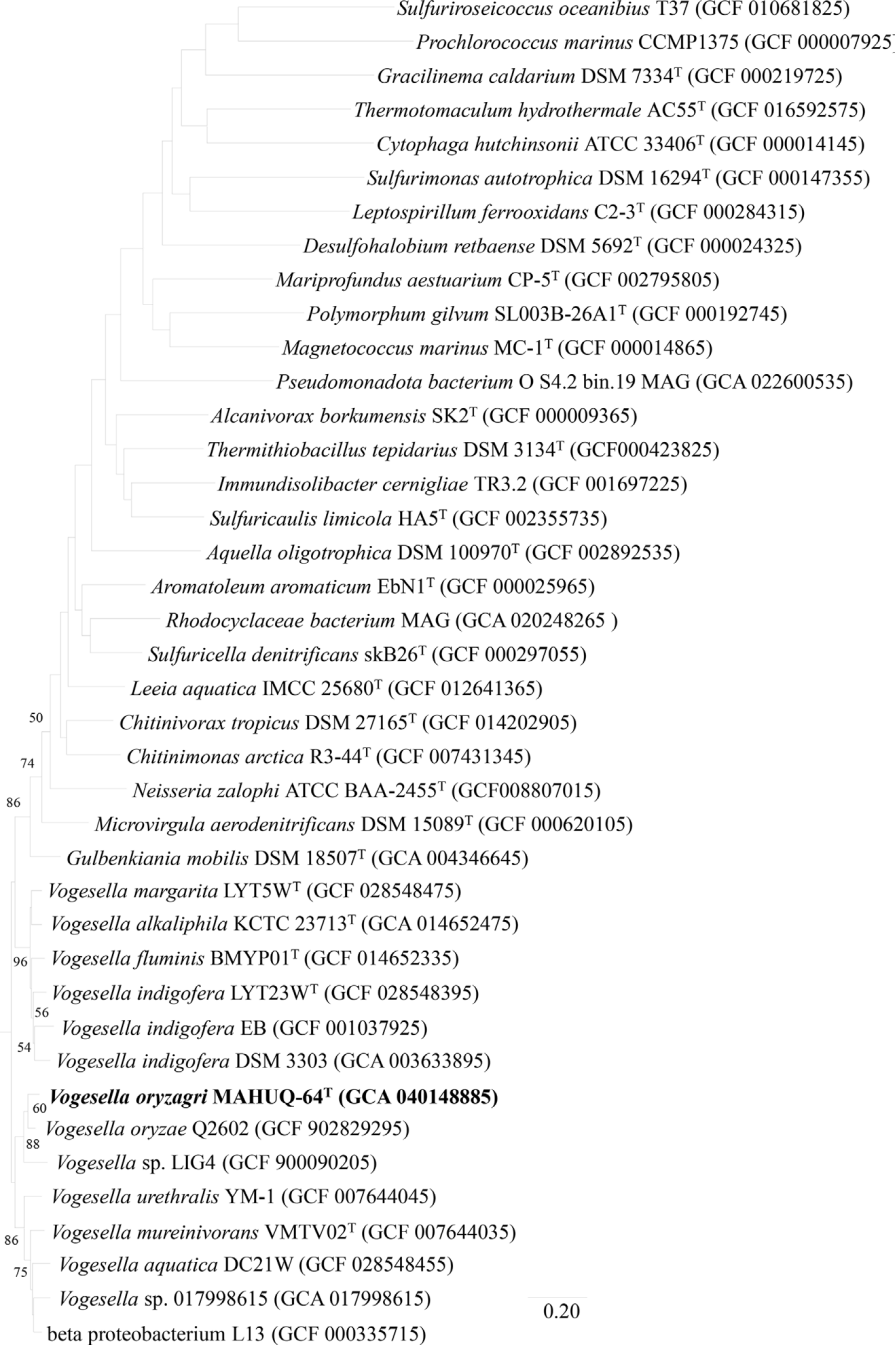
Phylogenomic tree of *V. oryzagri* MAHUQ-64^T^ and related taxa reconstructed using OrthoFinder. The tree was inferred based on orthologous genes identified from whole-genome protein sequences. The bar represents 0.20 substitutions per nucleotide position.

According to the EzBioCloud server analysis, 16S rRNA gene sequence analyses indicated that the close members of strain MAHUQ-64^T^ are *Vogesella oryzae* L3B39^T^ (98.6%) and *Vogesella facilis* TTM-24^T^ (98.2%). Similarities with all other strains were less than 97.3%. The 16S rRNA gene sequence of strain MAHUQ-64^T^ is a continuous stretch of 1468 bp (NCBI GenBank accession number MW487996). The relationship between strain MAHUQ-64^T^ and other members was supported by the topology of the phylogenetic trees (Figs 1, S1 and S2). The NJ tree showed that strain MAHUQ-64^T^ is clustered within the genus *Vogesella* and formed a clade with *V. oryzae* L3B39^T^ and *V. facilis* TTM-24^T^ (Fig. 1). The NJ tree was also constructed using all the close strains including uncultured bacterium from the NCBI database (Fig. S1). The NJ tree was supported by the tree created by the ML algorithm (Fig. S2) with high bootstrap values. Moreover, the phylogenetic tree that was constructed from MLSA of whole-genome sequences showed that strain MAHUQ-64^T^ is clustered with the members of genus *Vogesella* and formed a monophyletic clade with *V. oryzae* L3B39^T^ (Fig. S3). The phylogenetic tree inferred with OrthoFinder also showed that strain MAHUQ-64^T^ is placed within the genus *Vogesella* and formed a clade with *V. oryzae* (Fig. 2). The phylogenetic analysis indicated that strain MAHUQ-64^T^ is clearly grouped within the genus *Vogesella*. The draft genome sequence of strain MAHUQ-64^T^ yielded a genome of 3.8 Mb in length after assembly, producing 22 contigs with an N50 value of 484 500. The total genome size is 3 827 146 bp. Gene prediction allowed the annotation of 3612 protein-coding genes with 74 tRNA and 4 rRNA genes. The genomic DNA G+C content of strain MAHUQ-64^T^, directly calculated from its genome sequence, was determined to be 63.4 mol%, which is in the range of the type species of the genus *Vogesella* [1,6]. The genome sequence features of the novel strain MAHUQ-64^T^ are shown in Table S2. The ANI value between strain MAHUQ-64^T^ and the closest type strain *V. oryzae* L3B39^T^ was 86.5% (Table S3). The dDDH value between strain MAHUQ-64^T^ and the closest type strain *V. oryzae* L3B39^T^ was 33.4% (Table S3). These ANI and dDDH values are well below the species threshold of 95–96 and 70%, respectively, suggesting that MAHUQ-64^T^ represents a novel species [26,28]. Based on dDDH results, ANI values, and phylogenetic analysis, it is evident that the isolated strain represents a novel species belonging to the genus *Vogesella*.

## Comparative genomic studies

For a whole genome-based taxonomic analysis, the genome sequence data were uploaded to the Type (Strain) Genome Server (TYGS), a free bioinformatics platform accessible at https://tygs.dsmz.de (accessed 01 July 2024). The Genome blast Distance Phylogeny (GBDP) approach was also used to construct a phylogenetic tree using TYGS [29,30]. GBDP phylogenetic tree was constructed using whole-genome sequences. The CGView (http://cgview.ca/) was used to generate a graphical representation of the blast result comparison of the available genomes to the genome of *V. oryzagri* MAHUQ-64^T^. Taxonomic and functional research of micro-organisms has increasingly relied upon genome-based data and methods. The distribution of genes in the genome of strain MAHUQ-64^T^ and the closest reference strain *V. oryzae* L3B39^T^ was investigated using the RAST server [31]. The ecological distribution and habitat preferences of the strain MAHUQ-64^T^ were analysed using the Protologger web tool (www.protologger.de). Protologger provides an automated and comprehensive platform for interpreting genomic data to assess taxonomic, functional and ecological traits of microbial strains [32].

**Fig. 3.**
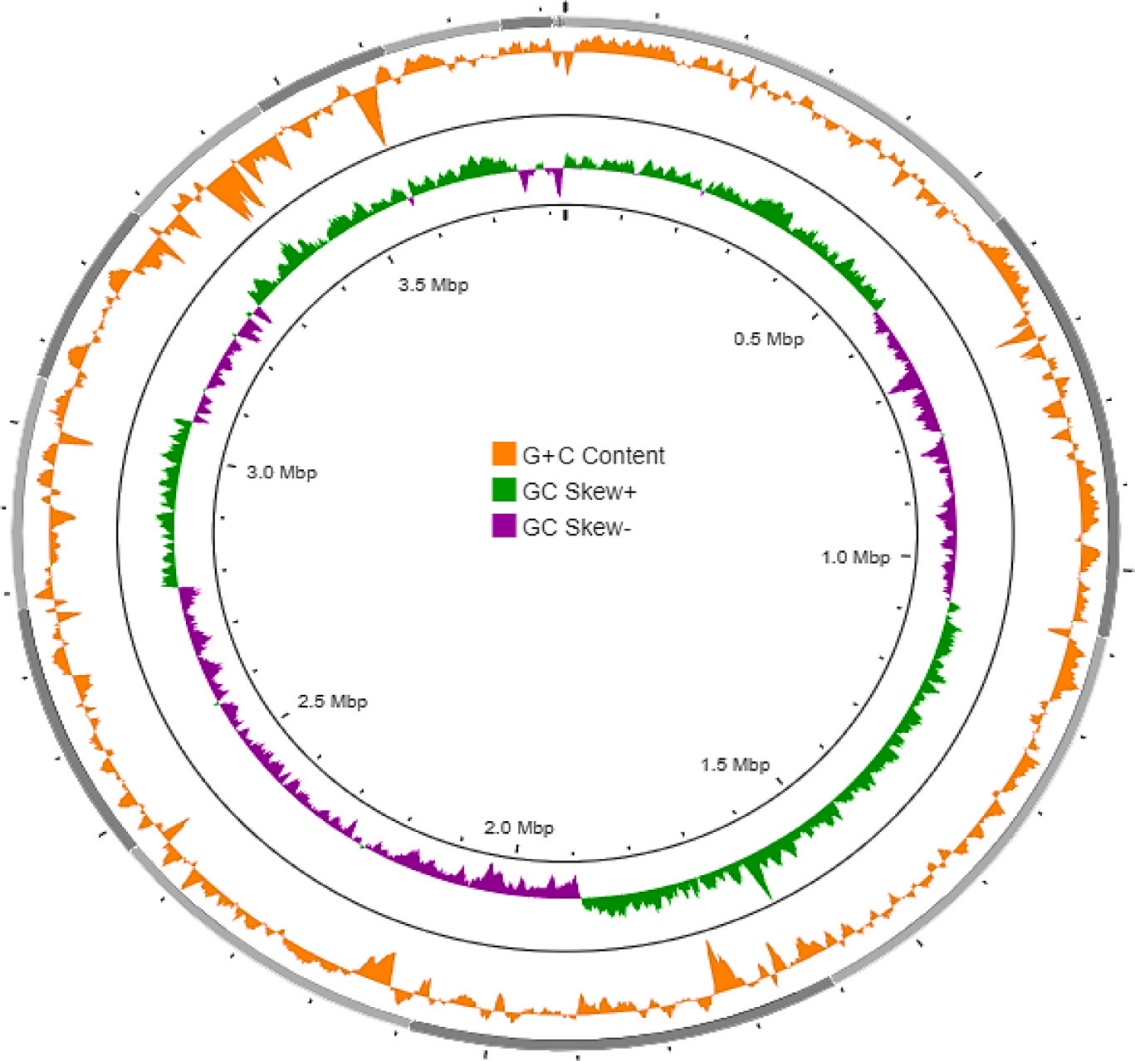
Schematic representation of the circular chromosome of novel strain MAHUQ-64^T^, created by CGView server (http://cgview. ca/http://cgview. ca/). Circle 1 (outermost) displays the contigs, circle 2 displays the G+C content plot and circle 3 (innermost) displays the GC skew. To indicate genome sizes inside and outside, the ruler was used in the chromosome map.

Using the GBDP method and tree builder service, the phylogenetic tree of strain MAHUQ-64^T^ using its whole-genome sequence was created. The GBDP phylogenetic tree constructed by using the whole genome indicated that strain MAHUQ-64^T^ clustered with the members of the genus *Vogesella* and formed a monophyletic clade with *V. oryzae* L3B39^T^ (Fig. S4). Fig. 3 shows the circular chromosomes based on the genome sequence of strain MAHUQ-64^T^ using the CGView server (http://cgview.ca/), which is a web-based tool for comparative genomics analysis on circular genomes [33]. The RAST functional annotations of the draft genome of strain MAHUQ-64^T^ showed that 198 of the genes were involved with protein metabolism, 336 genes were associated with the metabolism of amino acids and derivatives, 50 genes were involved with DNA metabolism, 149 genes were linked with carbohydrate metabolism and 142 genes were involved with the metabolism of vitamins, cofactors and pigments. Moreover, the genome of strain MAHUQ-64^T^ revealed 66 gene clusters for stress response and 99 genes for respiration (Table S4). The genome of strain MAHUQ-64^T^ has 97 genes for motility and chemotaxis (Table S4). The presence of genes for flagellar motility and the presence of flagella (Fig. S5) showed that the phenotypic and genomic results are consistent with each other. RAST functional analysis revealed that the genome of the closest type strain *V. oryzae* L3B39^T^ contains the same genes, but there were quantitative differences (Table S4). For example, the genome of strain MAHUQ-64^T^ contains 336 genes, but type strain *V. oryzae* L3B39^T^ contains 283 genes, which are responsible for aa and derivatives. Similarly, the genome of strain MAHUQ-64^T^ contains 97 genes, but type strain *V. oryzae* L3B39^T^ contains 36 genes, which are responsible for motility and chemotaxis (Table S4).

The 16S rRNA gene analysis of strain MAHUQ-64^T^ revealed 98.6% similarity to *V. oryzae* L3B39^T^, the closest valid species, indicating its novelty. Phylogenomic analysis, reconstructed using OrthoFinder and supported by high bootstrap values, placed the strain within the genus *Vogesella*. The analysis showed that all *Vogesella* strains formed a single cohesive cluster, reflecting their close evolutionary relationship. ANI analysis further confirmed the novelty of the strain, with the highest score of 86.5% to *V. oryzae*, well below the 95% species threshold. Additionally, the Percentage of Conserved Proteins (POCP) analysis yielded a value of 85.3% with *V. oryzae*. Since POCP values above 50% confirm genus-level affiliation, this strongly supports the classification of strain MAHUQ-64^T^ within the genus *Vogesella* [34]. Functional annotation predicted the presence of genes for acetate and propionate production from acetyl-CoA and propionoyl-CoA, vitamin biosynthesis pathways (B7, B12 and B9) and flagellar proteins, indicating metabolic versatility and motility. Ecological analysis using Protologger identified the strain’s prevalence in wastewater (7.0%), freshwater (18.1%) and soil (2.1%) environments. These results collectively confirm *V. oryzagri* MAHUQ-64^T^ as a novel species within the genus *Vogesella*.

## Secondary metabolite BGC prediction

As a main approach for finding and annotating genes in BGCs across the genome, antiSMASH 7 [35] combined with ClusterBlast, ActiveSiteFinder, Cluster Pfam analysis and SubClusterBlast [35] was used for the discovery of BGCs in the genome of *V. oryzagri* MAHUQ-64^T^ for secondary metabolites.

**Fig. 4.**
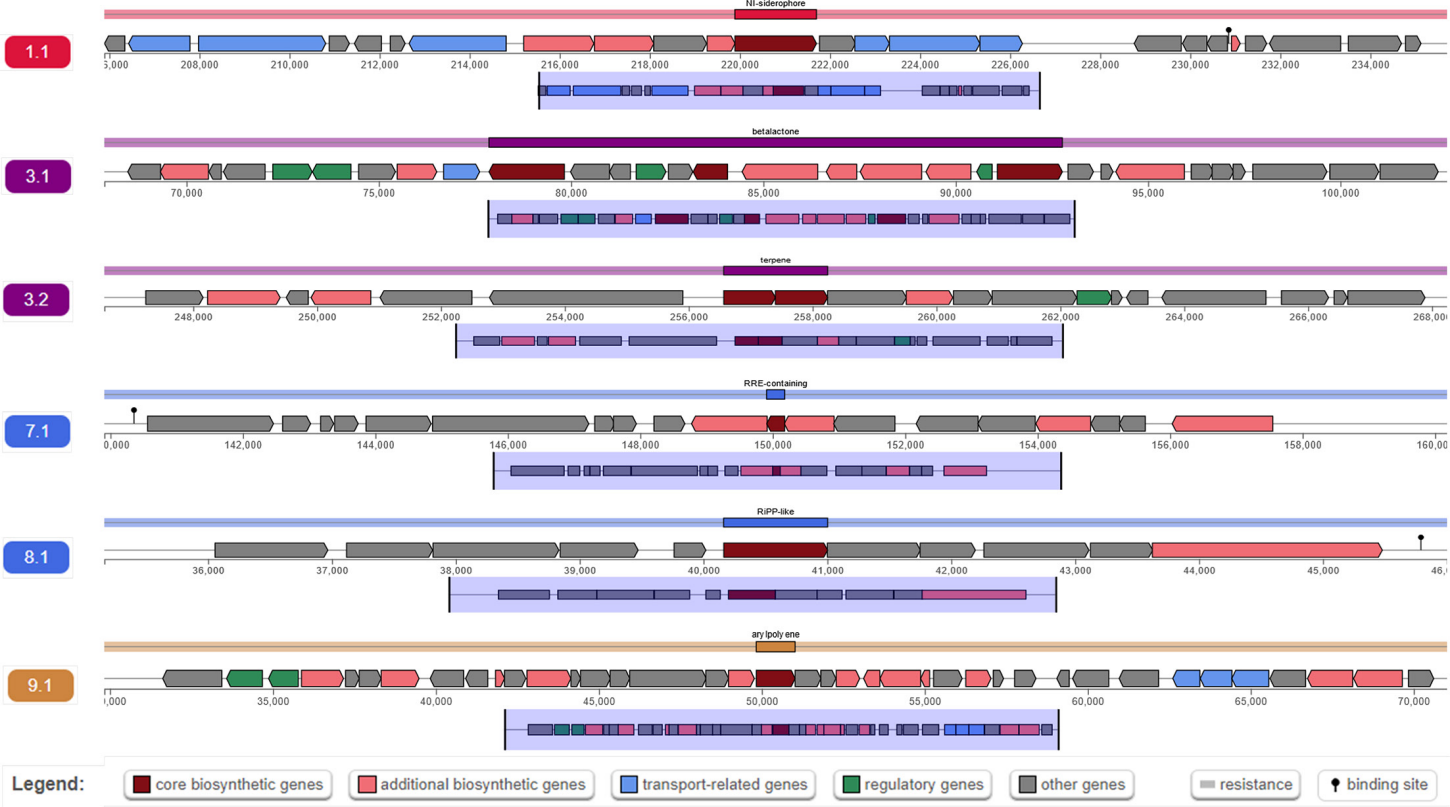
Localization of secondary metabolite clusters in the genome of *V. oryzagri* MAHUQ-64^T^.

Using antiSMASH 7.0, we found several BGCs on the genome of the novel strain *V. oryzagri* MAHUQ-64^T^ for different secondary metabolites. Through the prediction using antiSMASH 7.0, six BGCs were discovered in the genome of *V. oryzagri* MAHUQ-64^T^ (Table 1 and Fig. 4). The BGC types include those for non-ribosomal peptide synthetase independent (NI)-siderophore, betalactone, terpene, RiPP recognition element (RRE)-containing, Ribosomally synthesized and post-translationally modified peptide (RiPP)-like and arylpolyene were discovered in the genome of novel strain MAHUQ-64^T^ (Table 1 and Fig. 4). Among these BGCs, NI-siderophore (cluster 1, region 1.1) was 83% identical to known BGCs. Other BGCs exhibited a low degree of similarity or no similarity to previously identified BGCs, implying that the novel strain MAHUQ-64^T^ has a significant potential for the production of novel natural products in the future.

**Table 1.** The analysis of biosynthetic pathways in *V. oryzagri* MAHUQ-64^T^ by antiSMASH 7.0

Cluster serial no.	Region	Type	From	To	Most similar known cluster	**Similarity**
1	1.1	NI-siderophore	205 889	235 700	Fulvivirgamide A2, fulvivirgamide B2, fulvivirgamide B3 and fulvivirgamide B4	83%
2	3.1	Betalactone	67 865	102 771	Fengycin, NRP	13%
3	3.2	Terpene	246 565	268 243		
4	7.1	RRE-containing	139 908	160 180		
5	8.1	RiPP-like	35 163	46 002		
6	9.1	Arylpolyene	29 828	71 021	Gladiostatin A, NRP+polyketide	14%

## Physiology and chemotaxonomy

The Gram-stain reaction was determined using a bioMérieux Gram-stain kit according to the manufacturer’s instructions. The growth was tested using several bacterial media such as nutrient agar (NA) (Difco), tryptone soya agar (TSA) (Oxoid), R2A agar, Luria–Bertani (LB) agar (Oxoid) and MacConkey agar (Oxoid) at 30 °C. Growth at different temperatures (4, 10, 15, 20, 25, 28, 30, 35, 40 and 45 °C) and at various pH conditions (pH 3.0–10.0, at intervals of 0.5 pH units) was assessed in the R2A broth after 7 days of incubation at 30 °C. pH-dependent growth was tested using two different buffers (final concentration, 100 mM): acetate buffer was used for pH 3.0–6.5, and phosphate buffer was used for pH 7.0–10.0. Salt tolerance was tested in R2A broth supplemented with 0–5.0% (w/v) NaCl (at 0.5% intervals) after 7 days of incubation at 30 °C. Growth was estimated by monitoring the optical density at 600 nm. To check anaerobic growth, strain MAHUQ-64^T^ was cultured on R2A plates and incubated in a closed chamber with the AnaeroGen kit (Thermo Scientific), which was used for the generation of an anaerobic atmosphere for 14 days at 30 °C [36]. Cell morphology was observed at ×11 000 magnification with a transmission electron microscope (model JEM1010; JEOL) using cells grown for 3 days at 30 °C on R2A agar. Motility was assayed on the sulphide-indole-motility medium (Difco) [37]. Production of flexirubin-type pigments was determined by the procedures outlined by Fautz and Reichenbach [38]. Catalase activity was determined by bubble production in 3% (v/v) H_2_O_2_, and oxidase activity was determined using 1% (w/v) *N*, *N*, *N*′, *N*′-tetramethyl-1,4-phenylenediamine reagent as described by Huq [39]. Hydrolysis of the following substrates was tested using R2A agar as the basal medium: casein (2.0% skim milk, Oxoid), 1.0% starch (Difco), 0.1% aesculin (0.02% ferric citrate, Difco), Tween 80 (0.01% CaCl_2_.2H_2_O and 1.0% Tween 80, Sigma), Tween 20 (0.01% CaCl_2_.2H_2_O and 1.0% Tween 20, Sigma), 0.5% l-tyrosine (Sigma), 12.0% gelatin (Sigma) and DNA [DNase agar, Scharlau (Spain); DNase activity was revealed by flooding the plates with 1 N HCl]. Activities were evaluated after 7 days of incubation at 30 °C. For the comparative study, the reference strains *V. oryzae* L3B39^T^ and *V. facilis* TTM-24^T^ were included and tested using the same laboratory conditions. Carbon-source utilization and constitutive enzyme activities of strain MAHUQ-64^T^ and the reference strains were tested using API ZYM and API 20 NE test kits, according to the manufacturer’s instructions (bioMérieux, France).

Strain MAHUQ-64^T^ was aerobic, Gram-stain-negative, catalase and oxidase positive, motile, flagellated and rod-shaped (0.7–1.3 µm width and 1.5–2.8 µm long) (Fig. S5). The colonies were observed to be creamy white-coloured, smooth, spherical and 0.5–1.1 mm in diameter when grown on R2A agar medium for 2 days (Fig. S6). Growth occurred at 10–40 °C (optimum 30 °C) and at pH 5.0–9.5 (optimum 7.0). In the R2A broth medium, growth occurred in the presence of 0–2.0% (w/v) NaCl (optimum 0–0.5%). Strain MAHUQ-64^T^ was positive for the following enzyme activities: alkaline phosphatase, acid phosphatase, leucine arylamidase, valine arylamidase, cysteine arylamidase, naphthol-AS-BI-phosphohydrolase and esterase lipase (C8). Phenotypic and biochemical examinations revealed that strain MAHUQ-64^T^ shared several traits in common with its closest relatives. However, there are some morphological, physiological and biochemical differences between strain MAHUQ-64^T^ and phylogenetically closest related species that differentiate the novel strain MAHUQ-64^T^ from related type strains (Table 2). For example, strain MAHUQ-64^T^ can be easily differentiated from the phylogenetically close reference strains by indole production, DNA degradation, esterase and *N*-acetyl-*β*-glucosaminidase activity, assimilation ability of *N*-acetyl-glucosamine, d-maltose, capric acid and trisodium citrate and different growth conditions. The physiological, morphological and biochemical characteristics of strain MAHUQ-64^T^ and closest reference strains are summarized in Table 2 and the species description.

**Table 2.** The biochemical and physiological characteristics of strain MAHUQ-64^T^ and the closest reference strains Strains: 1, *V. oryzagri* MAHUQ-64^T^; 2, *V. oryzae* L3B39^T^; and 3, *V. facilis* TTM-24^T^. All data were obtained in this study, except ^a^ and ^b^ which were taken from Rameshkumar *et al.* [5] and Sheu *et al.* [6], respectively. All strains are rod-shaped and motile. All strains are positive for catalase, oxidase, acid phosphatase, reduction of nitrate and assimilation of d-glucose, l-arabinose, gluconate and malic acid. All strains are negative for fermentation of glucose, lipase (C14), *α*-mannosidase, *α*-glucosidase, *α*-galactosidase, *β*-glucosidase, *β*-galactosidase, *α*-fucosidase, hydrolysis of starch, casein, urea, aesculin, gelatin and assimilation of d-mannose, d-mannitol, adipic acid and phenylacetic acid. +, positive; W, weakly positive; −, negative.

Characteristics	1	2	3
Isolation source	Rice rhizosphere soil	Rhizosphere of rice	Freshwater river
Colony colour	Creamy white	Creamy	Colourless to white
Aerobic/facultative anaerobic	Aerobic	Aerobic	Facultative anaerobic
Indole production (API 20 NE)	+	−	+
Growth temperature (optimum, °C)	10–40 (30)	10–42 (28–30)^a^	15–37 (25)^b^
NaCl tolerance (optimum, %)	0–2.0 (0–0.5)	0–1.5 (0.5)^a^	0–1.0 (0.5)^b^
**Hydrolysis of:**			
DNA	−	−	+
**Enzyme activity (API ZYM):**			
Esterase (C4)	−	w	+
Alkaline phosphatase	+	w	+
Esterase lipase (C8)	w	w	+
Leucine arylamidase	+	w	+
Valine arylamidase	w	w	+
Naphthol-AS-BI-phosphohydrolase	w	+	+
*N*-Acetyl-*β*-glucosaminidase	−	w	+
**Assimilation of (API 20 NE):**			
*N*-Acetyl-glucosamine	+	−	+
d-maltose	−	+	−
Capric acid	−	+	+
Triosodium citrate	−	+	−
DNA G+C content (mol%)	63.4	62.3	67.4^b^

For fatty acid methyl ester analysis, cells of strain MAHUQ-64^T^ and the reference strains were harvested from R2A agar plates after 2 days of incubation, and fatty acids were determined as described by Sasser [40]. The isoprenoid quinones of strain MAHUQ-64^T^ were extracted from freeze-dried cell material. Quinones were extracted with chloroform/methanol (2 : 1, v/v), and extracts were evaporated under vacuum. The crude hexane–quinone solution was purified using Sep-Pak Vac silica cartridges (Waters) and subsequently analysed using an RP-HPLC system (Alliance 2690 system) [column; SunFireTM C18 (4.6×250 mm×5 µm), solvent; methanol/2-propanol (7：5, v/v), flow rate; 1.0 ml min^−1^] [41,42].

The fatty acid profiles of strain MAHUQ-64^T^ and the related type strain are shown in Table 3. The major cellular fatty acids of strain MAHUQ-64^T^ were identified as summed feature 3 (comprising C_16  :  1_*ω*7*c* and/or C_16  :  1_*ω*6*c*, 53.5%) and C_16  :  0_ (27.6%). Strain MAHUQ-64^T^ showed a similar major fatty acid composition to the closely related type strains, but there were quantitative differences when cultivated under the same conditions (Table 3). The major respiratory quinone of strain MAHUQ-64^T^ was identified as Q-8, which is one of the characteristics of members of the genus *Vogesella* [1,6].

**Table 3.** Fatty acid profiles of strain MAHUQ-64^T^ and the closest reference strains Strains: 1, *V. oryzagri* MAHUQ-64^T^; 2, *V. oryzae* L3B39^T^; and 3, *V. facilis* TTM-24^T^. All data were obtained from this work. Fatty acid percentages amounting to less than 1% of the total fatty acids in all strains were not included in the table.

Fatty acid	1	2	3
C_10 : 0_	3.9	2.7	3.4
C_10 : 0_ 3OH	1.7	2.0	2.8
C_12 : 0_	3.1	3.2	3.1
C_12 : 0_ 3OH	1.3	1.9	3.0
C_14 : 0_	2.4	2.8	2.2
C_16 : 0_	27.6	29.9	26.5
Sum in feature 3	53.5	54.6	51.3
Sum in feature 8	2.9	1.5	4.9

Sum in feature 3=C_16:1_ *ω*7*c* and/or C_16:1_ *ω*6*c*.

Sum in feature 8=C_18:1_ *ω*7*c* and/or C_18:1_ *ω*6*c*.

In summary, as indicated by the phylogenetic tree analysis, strain MAHUQ-64^T^ belongs to the genus *Vogesella*. In addition, the characteristics of strain MAHUQ-64^T^ are consistent with descriptions of the genus *Vogesella* with regard to morphological, biochemical and chemotaxonomic properties. However, strain MAHUQ-64^T^ can be distinguished from the closely related type strains not only by physiological and biochemical characteristics but also by low dDDH values and ANI values. The results of this polyphasic comparison between strain MAHUQ-64^T^ and its close phylogenetic neighbours indicated that strain MAHUQ-64^T^ should be assigned to the genus *Vogesella* as a novel species, for which the name *V. oryzagri* sp. nov. is proposed.

## Description of *Vogesella oryzagri* sp. nov.

*Vogesella oryzagri* (o.ryz.a'gri. L. fem. n. *oryza*, rice; L. masc. n. *ager*, a field; N.L. gen. n. *oryzagri*, of a rice field).

Cells are Gram-stain-negative, aerobic, motile, flagellated and rod-shaped (0.7–1.3 µm wide and 1.5–2.8 µm long). Colonies on R2A agar medium are smooth, spherical, creamy white-coloured and 0.5–1.1 mm in diameter when grown for 2 days. They are positive for both catalase and oxidase tests. Flexirubin-type pigments are absent. Growth occurs on TSA, NA, LB agar and R2A agar. Growth occurs at 10–40 °C (optimum 30 °C) and at pH 5.0–9.5 (optimum 7.0). In R2A broth medium, growth occurs in the presence of 0–2.0% (w/v) NaCl (optimum 0–0.5%). Cells can hydrolyze l-tyrosine and l-arginine, but not Tween 80, Tween 20, urea, starch, aesculin, casein, gelatin and DNA. The tests for nitrate reduction and indole production are positive, but glucose fermentation is negative. Strain MAHUQ-64^T^ is positive for the following enzyme activities: alkaline phosphatase, acid phosphatase and leucine arylamidase; weakly positive for esterase lipase (C8), valine arylamidase and cysteine arylamidase; but negative for lipase (C14), trypsin, *N*-acetyl-*β*-glucosaminidase, esterase (C4), *α*-chymotrypsin, *β*-glucuronidase*, α*-mannosidase, *β*-glucosidase, *β*-galactosidase, *α*-glucosidase, *α*-fucosidase and *α*-galactosidase (API ZYM). d-Glucose, l-arabinose, *N*-acetyl-glucosamine, gluconate and malic acid are utilized as sole carbon sources, but the following compounds are not utilized as sole carbon sources: d-mannitol, d-mannose, d-maltose, adipic acid, capric acid, phenylacetic acid and triosodium citrate (API 20 NE). The predominant isoprenoid quinone is Q-8. The major fatty acids are summed feature 3 (comprising C_16  :  1_*ω*7*c* and/or C_16  :  1_*ω*6*c*) and C_16  :  0_. The genomic DNA G+C content is 63.4 mol%.

The type strain, MAHUQ-64^T^ (=KACC 22245^T^=CGMCC 1.19000^T^), was isolated from the rhizosphere of a rice field located in Anseong, South Korea.

The NCBI GenBank accession numbers for the 16S rRNA gene and draft genome sequences of MAHUQ-64^T^ are MW487996 and JBEFLD000000000, respectively.

## Supplementary material

10.1099/ijsem.0.006687 Uncited Supplementary Material 1.

## Abbreviations

ANI: average nucleotide identity
BGCs: biosynthetic gene clusters
CGMCC: China General Microbiological Culture Collection Center
dDDH: digital DNA–DNA hybridization
GBDP: Genome blast Distance Phylogeny
KACC: Korean Agricultural Culture Collection
LB: Luria–Bertani
ML: maximum-likelihood
NA: nutrient agar
NJ: neighbour-joining
POCP: Percentage of Conserved Proteins
Q-8: ubiquinone-8
R2A: Reasoner’s 2A
TSA: tryptone soya agar
TYGS: Type (Strain) Genome Server
