# Supplementary material for: Vogesella oryzagri sp. nov., isolated from the rhizosphere of rice and in silico genome mining for the prediction of biosynthetic gene clusters
Source: Int J Syst Evol Microbiol. 2025 Feb 26;75(2):006687. doi: 10.1099/ijsem.0.006687 (PMC12282012; doi:10.1099/ijsem.0.006687)
Supplement: Uncited Supplementary Material 1. [file ijsem-75-06687-s001.pdf]

Supplementary Material

Vogesella oryzagri sp. nov., isolated from the rhizosphere of rice and in silico genome mining for the prediction of biosynthetic gene clusters.

Md. Amdadul Huq[1*], Yeon-Ju Kim[2], M. Mizanur Rahman[3], Md. Morshedul Alam[4], Sathiyaraj Srinivasan[5], Kwon-Kyoo Kang[6], Shahina Akter[7]

[1] *Department of Food and Nutrition, College of Biotechnology and Natural Resource, Chung-Ang University, Anseong-si, Gyeonggi-do, 17546, Republic of Korea.*

[2] *Graduate School of Biotechnology, and College of Life Science, Kyung Hee University, Yongin-si, Gyeonggi-do 17104, Republic of Korea*

[3] *Department of Biotechnology and Genetic Engineering, Faculty of Biological Science, Islamic University, Kushtia-7003, Bangladesh.*

[4] *Department of Biochemistry and Microbiology, School of Health and Life Sciences, North South University, Dhaka 1229, Bangladesh.*

[5] *Department of Bio & Environmental Technology, College of Natural Science, Seoul Women's University, Seoul, 01797, Republic of Korea.*

[6] *Department of Horticultural Life Science, Hankyong National University, Anseong-si, Gyeonggi-do, 17579, Republic of Korea.*

[7] *Department of Food Science and Biotechnology, Gachon University, Seongnam, 461-701, Republic of Korea.*

[*]Corresponding author:

Md. Amdadul Huq, E-mail: amdadbge@gmail.com, amdadbge100@cau.ac.kr

Supplementary Fig. S1. Neighbor-Joining (NJ) phylogenetic tree based on 16S rRNA gene sequences showing the position of *Vogesella oryzagri* MAHUQ-64^{T} and other strains including uncultured bacterium. Bootstrap values more than 70 % based on 1,000 replications are shown at branching points. Scale bar, 0.002 substitutions per nucleotide position.

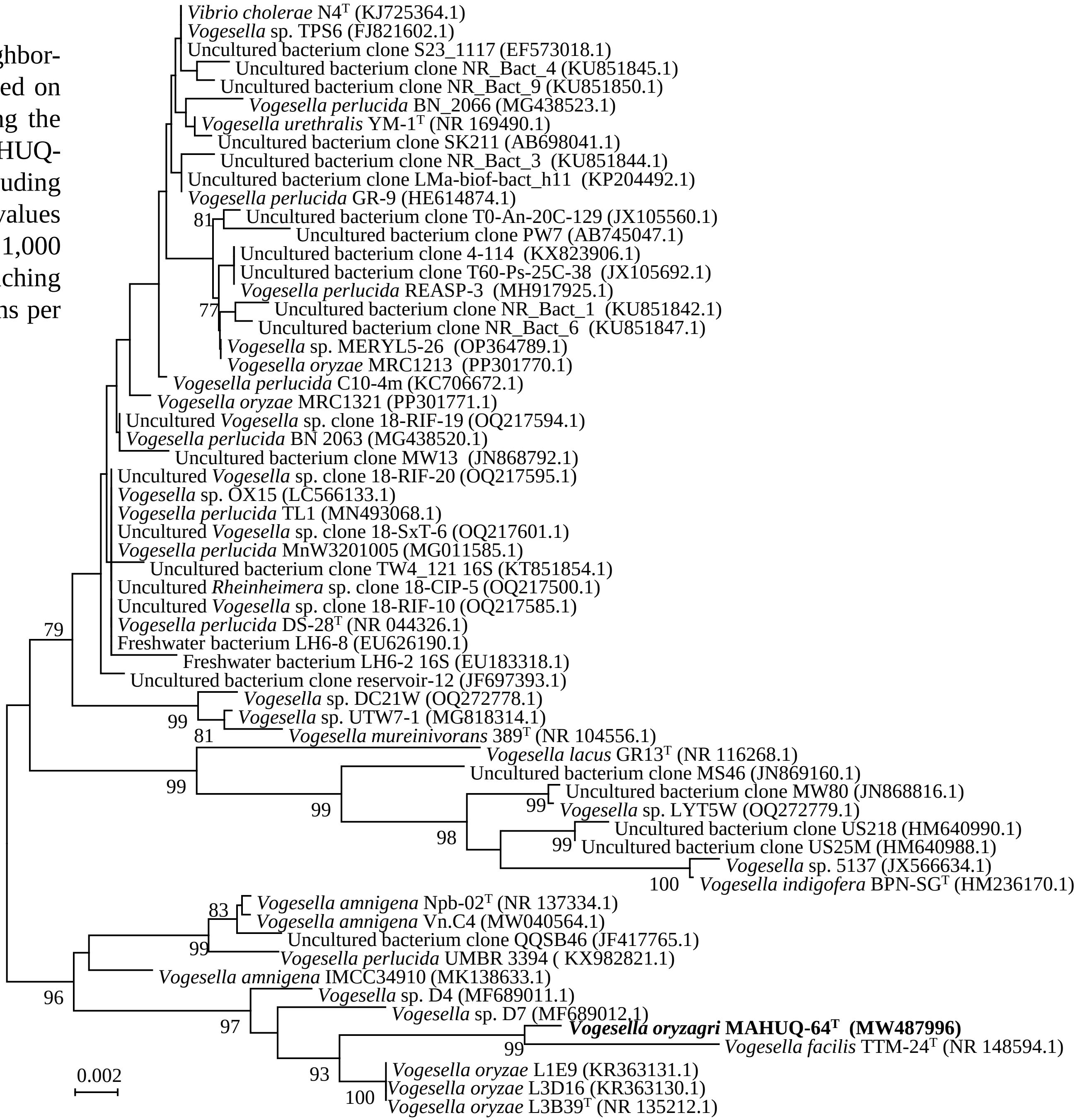

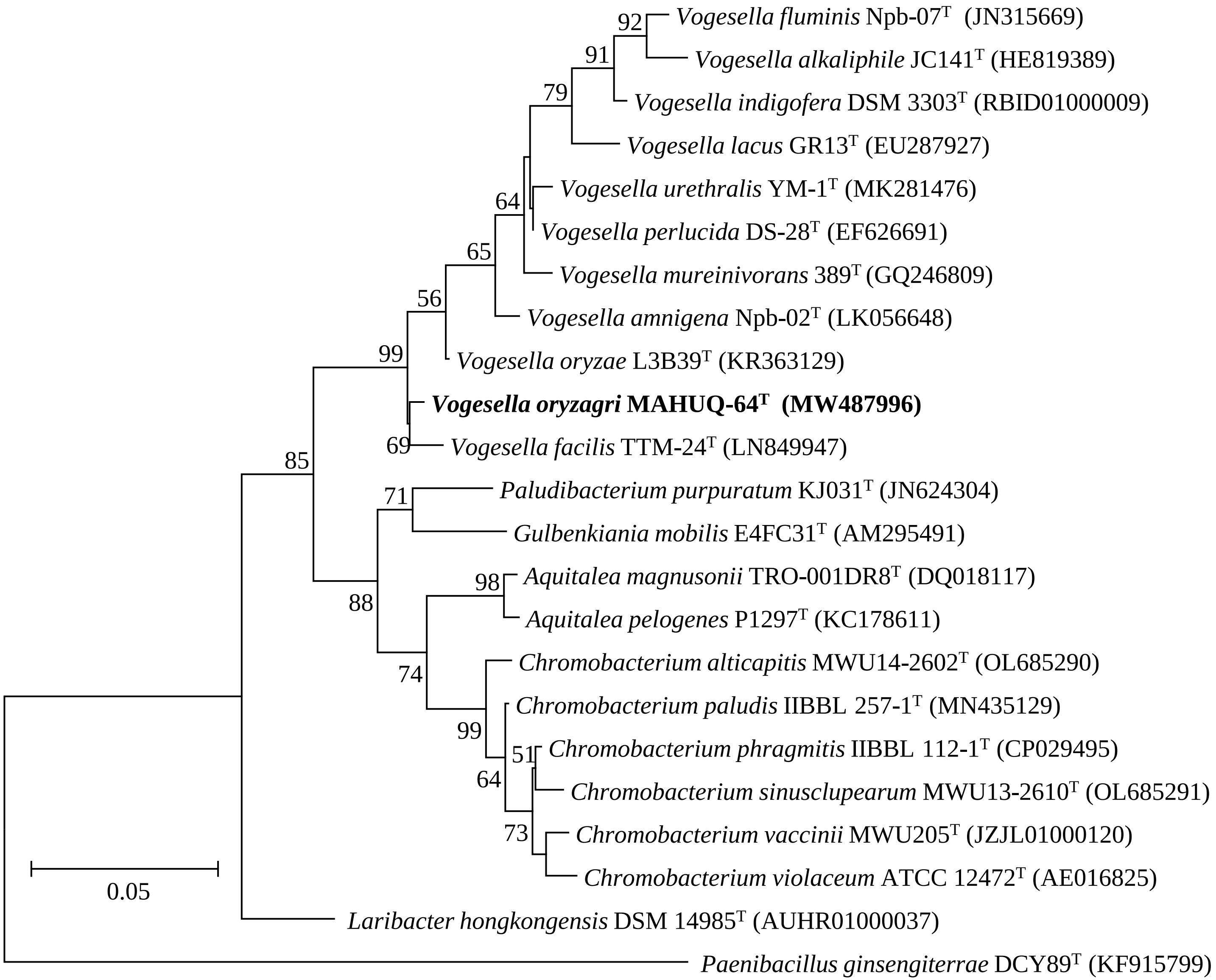

Supplementary Fig. S2. Maximum-likelihood (ML) phylogenetic tree based on 16S rRNA gene sequences showing the position of *Vogesella oryzagri* MAHUQ-64^T and other related species. Bootstrap values less than 50 % based on 1,000 replications are not shown at branching points. *Paenibacillus ginsengiterrae* DCY89^T was used as an outgroup. Scale bar, 0.05 substitutions per nucleotide position.

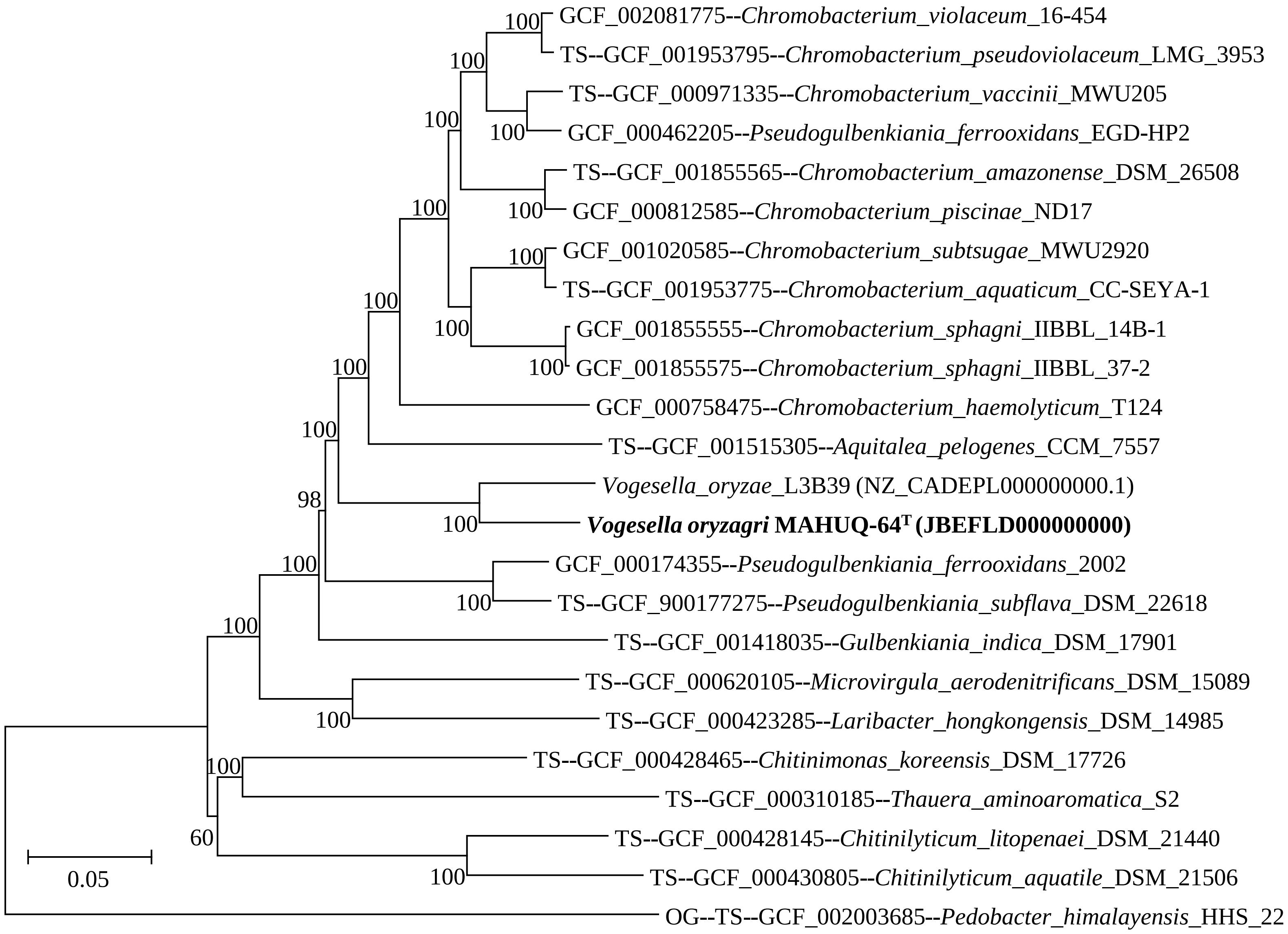

Supplementary Fig. S3. Phylogenetic tree constructed from a comparative analysis of whole genome sequences showing the relationships of strain MAHUQ-64^T with other closest species. This tree was constructed via Automated Multi-Locus Species Tree online web server, and with Mega-7 program using the aligned sequences of Automated Multi-Locus Species analysis. Bootstrap values (expressed as percentages of 1000 replications) greater than 50 % are shown at the branch points. The Bar represents 0.05 substitutions per nucleotide position.

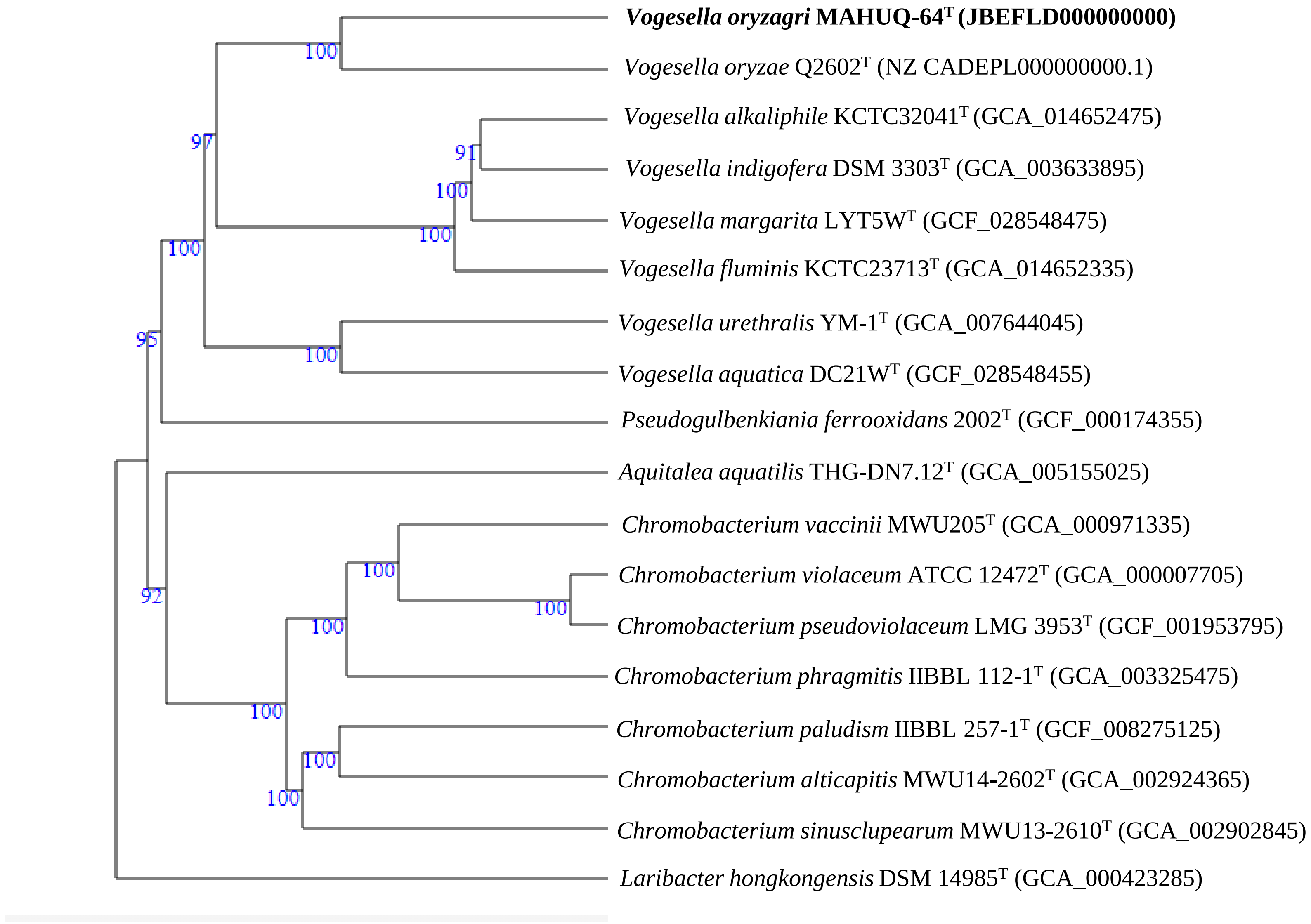

Supplementary Fig. S4. Whole-genome sequence-based GBDP (Genome BLAST Distance Phylogeny) tree showing the relationships of strain MAHUQ-64^T with other closest type species. Tree inferred with FastME 2.1.6.1 from GBDP distances calculated from genome sequences. The branch lengths are scaled in terms of GBDP distance formula d5. The numbers above branches are GBDP pseudo-bootstrap support values > 60 % from 100 replications, with an average branch support of 98.3 %. The tree was rooted at the midpoint.

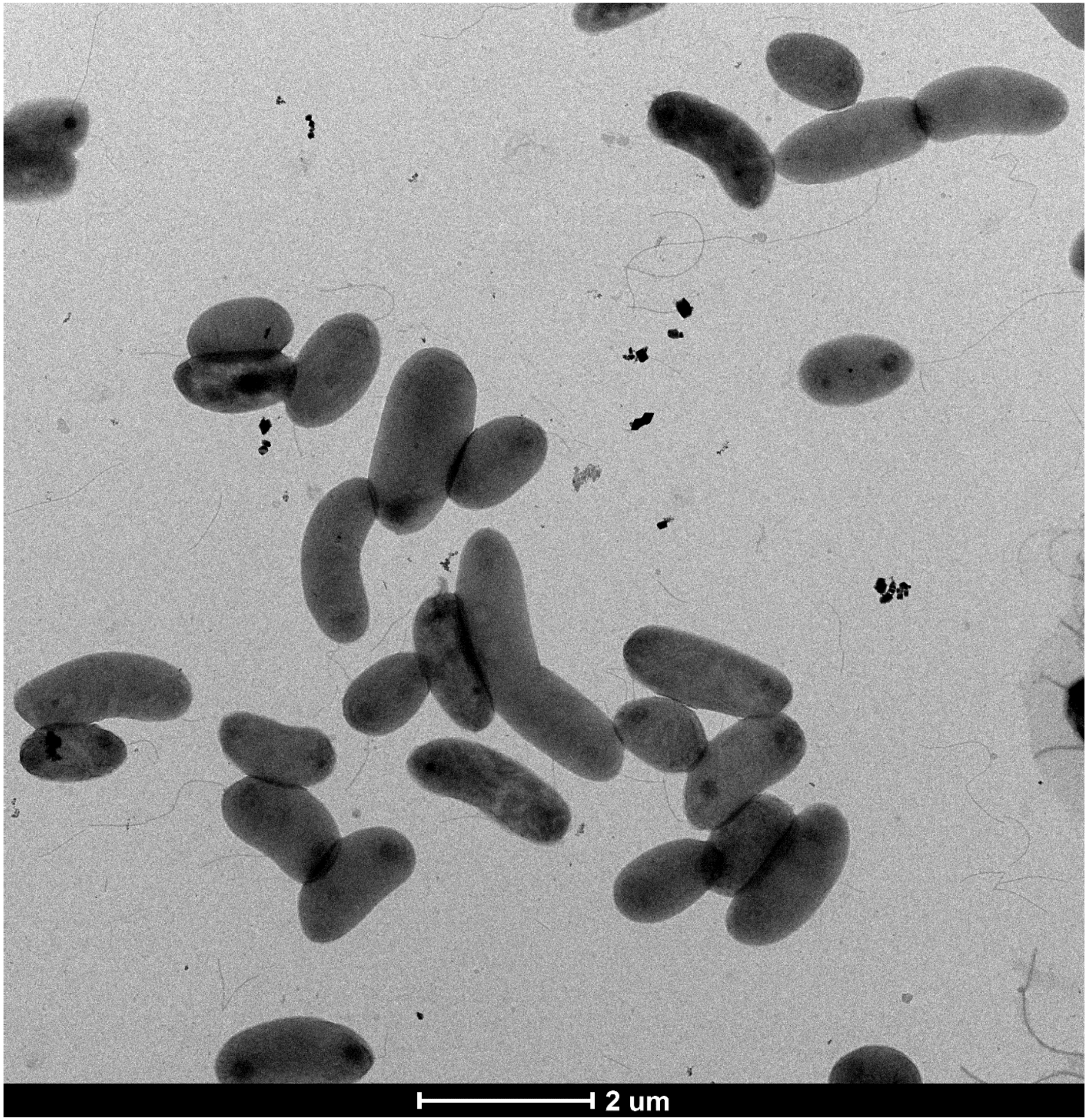

Supplementary Fig. S5. Transmission electron micrograph of *Vogesella oryzagri* MAHUQ-64^{T} after negative staining with uranyl acetate.

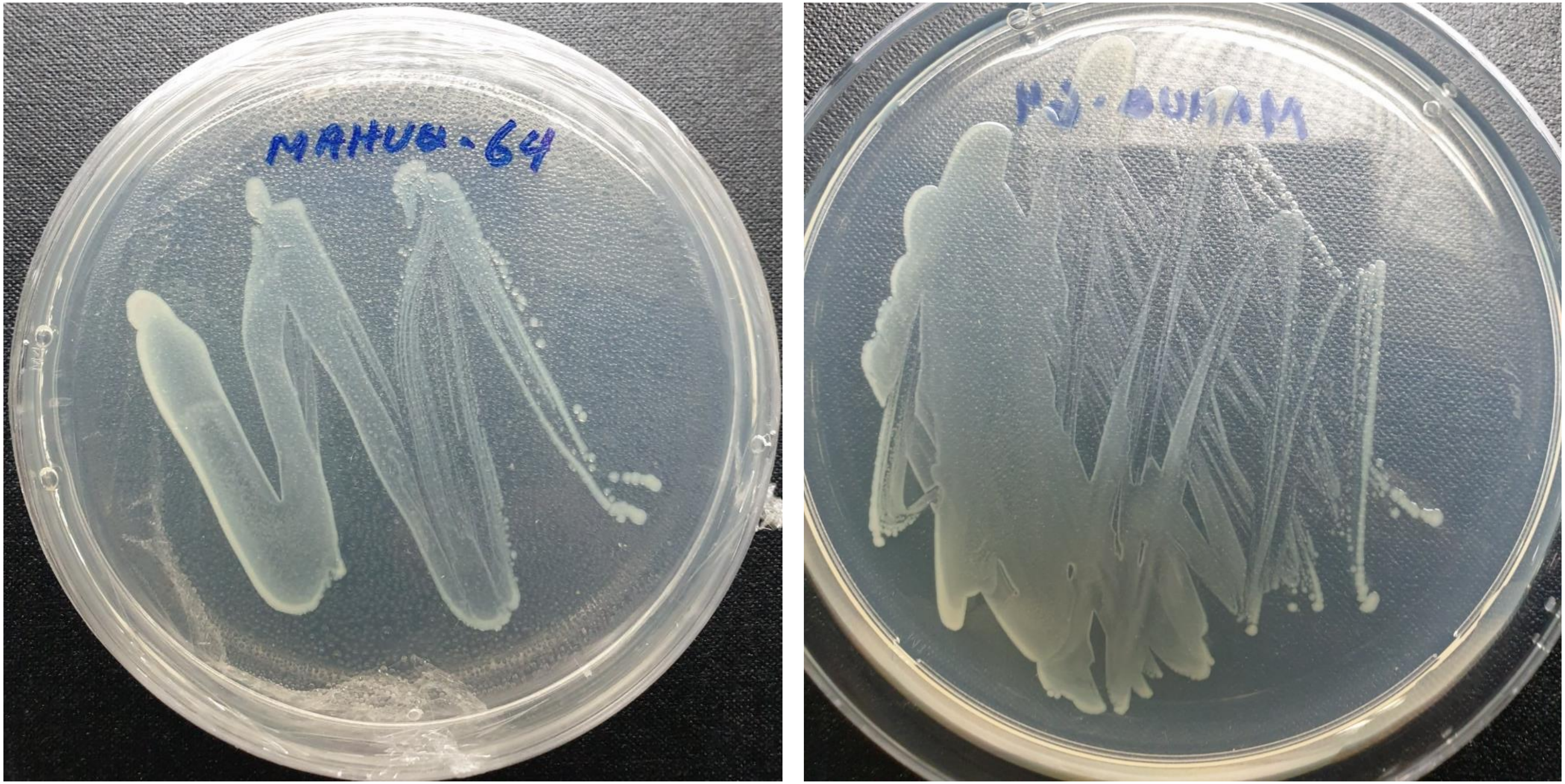

Supplementary Fig. S6. *Vogesella oryzagri* MAHUQ-64^T on R2A agar medium after 2 days of incubation at 30 °C.

Supplementary Table S1. Bacterial strains isolated from rice field.

Strain name	NCBI Accession number	Closest type species	16s rRNA gene sequence similarity (%)
MAHUQ-64^{T}	MW487996	*Vogesella oryzae* L3B39^{T}	98.6
M.A.Huq-107	PQ276986	*Lysinibacillus fusiformis* NBRC 15717^{T}	99.7
M.A.Huq-108	PQ276987	*Priestia megaterium* NBRC 15308^{T}	100
M.A.Huq-109	PQ276988	*Rossellomorea marisflavi* JCM 11544^{T}	99.7
M.A.Huq-110	PQ276989	*Bacillus velezensis* CR-502^{T}	99.9

Supplementary Table S2. Genome sequence features of novel strain *Vogesella oryzagri* MAHUQ-64^T.

**Features**	**Strain MAHUQ-64^T**
NCBI Accession No.	JBEFLD000000000
Biosample	SAMN41698439
BioProject	PRJNA1120520
Genome coverage	156.0x
Completeness	99.78%
Contamination level	0.22%
Total sequence length (nt)	3,827,146
Number of contigs	22
Number of scaffolds	17
Scaffold N50	484,500
Scaffold L50	4
Sequencing method	de novo
Annotation pipeline	NCBI Prokaryotic Genome
DNA G+C content (%)	63.4
Total genes	3,705
Genes (coding)	3,612
Number of RNAs	82
tRNAs	74
rRNAs	4

Supplementary Table S3. dDDH and ANI values between the proposed novel strain *Vogesella oryzagri* MAHUQ-64^T and the closest type strain *Vogesella oryzae* L3B39^T.

**Query genome**	**Reference genome**	**dDDH value**				**ANI (%)**
		DDH	Model C.I.	Distance	Prob. DDH >= 70%	
Vogesella oryzagri MAHUQ-64^T (JBEFLD000000000)	*Vogesella oryzae* L3B39^T (NZ_CADEPL000000000.1)	33.4%	[31 - 36%]	0.1250	0.39%	86.5

Supplementary Table S4.

Distribution of genes based on COG functional categories in the genomes of novel strain *Vogesella oryzagri* MAHUQ-64^T and the closest type strain *Vogesella oryzae* L3B39^T.

COG Function	*Vogesella oryzagri*	*Vogesella oryzae*
Cofactors, vitamins, prosthetic groups, pigments	142	135
Cell wall and capsule	31	29
Virulence, disease and defense	33	32
Potassium metabolism	10	8
Photosynthesis	0	0
Miscellaneous	29	28
Phages, prophages, transposable elements, plasmids	2	0
Membrane transport	39	51
Iron acquisition and metabolism	3	3
RNA metabolism	46	45
Nucleosides and nucleotides	71	61
Protein metabolism	198	197
Cell division and cell cycle	0	0
Motility and chemotaxis	97	36
Regulation and cell signaling	24	20
Secondary metabolism	5	4
DNA metabolism	50	53
Fatty acids, lipids, and isoprenoids	42	45
Nitrogen metabolism	35	35
Dormancy and sporulation	1	1
Respiration	99	96
Stress response	66	55
Metabolism of aromatic compounds	31	11
Amino acids and derivatives	336	283
Sulfur metabolism	6	5
Phosphorus metabolism	17	17
Carbohydrates	149	120
